# Effectiveness and Safety of Acupuncture for Poststroke Dysphagia: Study Protocol for a Pragmatic Multicenter Nonrandomized Controlled Trial

**DOI:** 10.1155/2017/2349794

**Published:** 2017-01-29

**Authors:** Yu Tat Chan, Hong Wei Zhang, Yuan Qi Guo, Zhi-Xiu Lin

**Affiliations:** ^1^School of Chinese Medicine, Faculty of Medicine, The Chinese University of Hong Kong, Shatin, Hong Kong; ^2^Pok Oi Hospital, The Chinese University of Hong Kong Chinese Medicine Centre for Training & Research, Shatin, Hong Kong

## Abstract

*Background. *Dysphagia is one of the most common complications of stroke. Acupuncture is widely employed to treat poststroke dysphagia in East Asia. No evidence is established to support such treatment approach. This proposed study aims to evaluate the effectiveness and safety of acupuncture for the treatment of poststroke dysphagia.* Methods and Design.* This is a multicenter, pragmatic, single-blinded, nonrandomized controlled clinical trial. A total of 140 eligible patients will be enrolled in the study. Subjects who are eligible in study but refuse to have acupuncture treatment will be put on the no-acupuncture control arm. Both groups of patients will receive standard routine care, while the patients of intervention group will receive add-on standardized acupuncture treatment. Each participant in intervention group will receive a total of 24 sessions of acupuncture treatment (three times per week). The primary outcome measure is the Royal Brisbane Hospital Outcome Measure for Swallowing (RBHOMS). Secondary outcome measures include functional oral intake scale, swallow quality-of-life questionnaire in Chinese version, BMI of the participant, and adverse events. All outcome measures will be assessed at baseline, at the end of acupuncture treatment (month 2), and at two months after treatment (month 4).* Ethics and Dissemination.* The ethics approval of clinical research study was granted by the Research Ethics Committee of both New Territories East and West Cluster of Hong Kong. Written informed consent will be obtained from all participants and the study will be undertaken according to the ICH-GCP Guidelines.* Trial Registration.* This trial is registered with chictr.org (registration number: ChiCTR-TRC-12002621 and registration date: 2012-10-26).

## 1. Background

Dysphagia, sometimes as part of pseudobulbar palsy, is one of the most common complications of stroke among patients. The prevalence of poststroke dysphagia ranges widely from 29% to 81% due to various methods of diagnosis, time after stroke, and the types of lesions [1–3]. Dysphagia may naturally resolve in many patients over the first 7 days after the cerebrovascular event and about 10% of them may develop swallowing problems six months after stroke [4, 5]. Dysphagia is known to be associated with an increased risk of pneumonia, malnutrition, disability, and mortality [2, 6, 7]. Many dysphagia management options are available today, including texture-modified diets, swallowing therapy programmes, nonoral feeding, medications, and physical stimulation. However, clinical evidence to establish their roles in the management of poststroke dysphagia is limited [8, 9].

Acupuncture is widely employed in management of poststroke complication in Asia and Pacific region. According to a report which was published by World Health Organization (WHO) in 2003, stroke is one of indications of acupuncture (Chmielnicki, 2014) [10]. Moreover, some clinical trials have been conducted to evaluate the effectiveness and safety of acupuncture for poststroke dysphagia. A study conducted by Ding et al. 1996 compared the acupuncture group and conventional western medication treatment. It demonstrated that the average recovery time of acupuncture group is shorter than conventional western medication group [11].

A systematic review which included about 35 randomized controlled trials of acupuncture for dysphagia has shown that acupuncture may improve swallowing function [12]. Some mechanistic studies had also illustrated that acupuncture is able to regulate the cortex and the swallowing center of the reticular structure of the brain stem to control swallowing reflection and coordinate motor movement of the swallow-related muscles, as well as directly improving the recovery of the injured peripheral nerves [13, 14]. Other studies had also found that acupuncture treatment could attenuate the plasma endothelin and nitric oxide (NO) level, regulate the imbalance between prostacyclin and thromboxane A2, and improve blood viscosity, thereby collectively contributing to the therapeutic effect for poststroke dysphagia [15–17].

Nevertheless, several limitations of these studies may potentially bias the result and render the findings inconclusive. Xie et al. had undertaken a systematic review which was published in Cochrane Library [18], demonstrating that there is no enough evidence to make any conclusion about treatment effect of poststroke dysphagia. Some flaws in methodology existed in the previous study to hinder the conclusive finding, such as small sample size and lack of blinding in assessment [19]. In addition, most previous studies are open-labelled trials. Blinding was usually not applied to the outcome assessors. The lack of blinding would seriously weaken the credibility of the study. Therefore, a high quality and well-designed randomized controlled trial is urgently required.

The previous studies, together with our previous experience, suggest that there is a need to conduct a well-designed pilot study for assessing the effectiveness and safety of acupuncture for poststroke dysphagia. The early conclusion of this pilot study is able to provide evidence for conducting a full-scale rigorously designed clinical trial in future.

## 2. Objective

This study is to evaluate the effectiveness and safety of acupuncture for the treatment of dysphagia as a complication of stroke.

## 3. Methods and Design

### 3.1. Study Design

**Figure 1:**
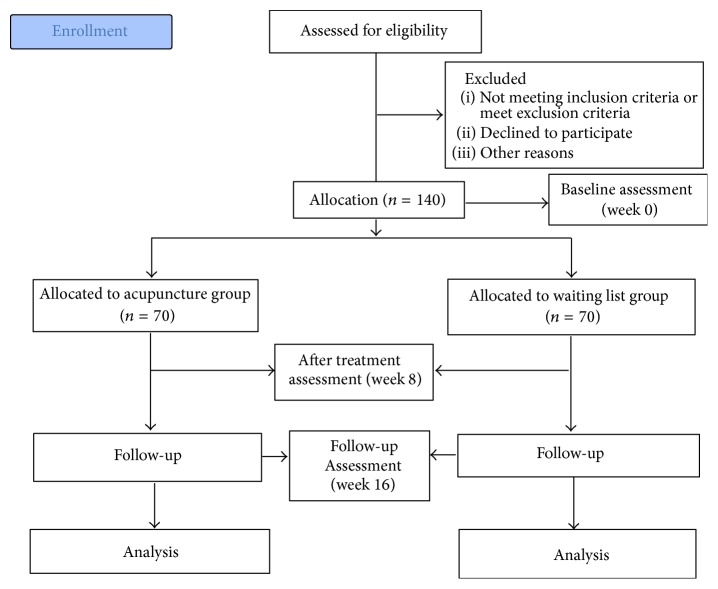
Study flow chart.

This is a multicenter, pragmatic clinical trial comparing real acupuncture intervention with no acupuncture to evaluate the treatment effects of acupuncture on the outpatient population of poststroke dysphagia. Owing to the characteristic of poststroke dysphagia patient and the nature of acupuncture, it is not feasible to implement the sham acupuncture in this study. The patients in acupuncture group and no-acupuncture control group will be in 1 : 1 allocation ratio. Subjects who are eligible in study and willing to accept acupuncture will be allocated to intervention group, while subjects eligible in study but refuse to have acupuncture treatment will be put on the no-acupuncture control arm. In the real acupuncture group, patients will receive acupuncture intervention in addition to conventional treatment. Real acupuncture group is defined as the group in the study that receives a series of sessions of acupuncture treatment which is being tested. The study will be conducted in several Chinese medicine clinics including The Pok Oi Hospital, The Chinese University of Hong Kong Clinical Center for Teaching and Research in Chinese Medicine (Shatin and Yuen Long), and The Yan Oi Tong, The Chinese University of Hong Kong Clinical Center for Teaching and Research in Chinese Medicine (Tuen Mun). Acupuncture treatment may also be conducted at the affiliated mobile clinics of these centers. This project is approved by both the Joint Chinese University of Hong Kong-New Territories East Cluster Clinical Research Ethics Committee (CRE-2012.236-T) and New Territories West Cluster Clinical Research Ethics Committee (NTWC/CREC/1281/14). Written informed consent is obtained from each participant. The study will be conducted according to ICH-GCP Guidelines. The study design is described in Figure 1.

### 3.2. Subject Recruitment

The patients may be recruited from The Pok Oi Hospital, The Chinese University of Hong Kong Clinical Center for Teaching and Research in Chinese Medicine (Shatin and Yuen Long), and The Yan Oi Tong, The Chinese University of Hong Kong Clinical Center for Teaching and Research in Chinese Medicine (Tuen Mun), as well as speech therapy clinics at Prince of Wales Hospital, Shatin Hospital, and Tuen Mun Hospital. The suitable poststroke dysphagia patients will be referred to by the speech therapists of the Speech Therapy Department from above hospitals. The Research Assistant will go to each of speech therapy outpatient clinics of all above three hospitals once or twice a week to explain to those potentially suitable patients (or their relatives/caretakers) the details of the research. All potential participants will be informed of the study such as objective, scope, procedure, and potential benefit and risks of the trial. A paper-formed voluntarily signed written inform consent will be obtained from participants. Under some circumstances, oral informed consent is acceptable, for instance, if participants cannot read. One hundred and forty eligible participants will be recruited from above sites according to the inclusion and exclusion criteria.

### 3.3. Inclusion Criteria

1. Age between 30–90 years old
2. With an ischemic or haemorrhagic stroke confirmed by a CT scan
3. Within 12 months after the first-time stroke (ischemic or haemorrhagic)
4. On modified diet due to dysphagia fulfilling RBHOMS with 2–8 points
5. Able to give informed consent (by patient himself/herself or by caretaker)

### 3.4. Exclusion Criteria

1. Dysphagia caused by head injury or neurological disease other than stroke
2. Patients who suffered from recurrent stroke (not the first-time stroke)
3. Unstable cardiac arrhythmia
4. Life-threatening infection
5. Unconsciousness or severe cognition deficits
6. Oral or throat diseases that display dysphagia
7. Patients with bleeding tendencies or thrombocytopenia, who are taking anticoagulants or have advanced malignancy
8. Patients with tracheostomy

### 3.5. Withdrawal Criteria

1. Participant's decision to drop out from study at any time for any reason
2. Noncompliance with the treatment protocol
3. Occurrence of any unexpected serious side effects

## 4. Blinding

Throughout the whole study process, the outcome assessor will be kept blind. As this is a single-blind controlled designed study, it is unfeasible to keep the patient and acupuncturist blind. The primary outcome measure will be carried out by registered speech therapist hired by the project. They will not be informed any information about the patient allocation and will be instructed to keep minimum interaction with patients during the whole study.

## 5. Intervention

Patients in both acupuncture and no-acupuncture control group will receive the conventional supportive and rehabilitative treatment, such as swallowing therapy programmes, nonoral feeding, medications, and physical stimulation [8, 9]. On top of the supportive treatment, patients in the acupuncture group will receive real acupuncture treatment, while patients assigned to the control group will receive no-acupuncture treatment during the study. Baseline assessment will be conducted for all eligible participants. The participants who agree to accept acupuncture treatment will be allocated to treatment group, while those who refuse to accept acupuncture treatment will be allocated to control arm.

All acupuncture treatment sessions will be performed by two to three acupuncturists who are registered Chinese Medicine Practitioners in Hong Kong and have at least 3 years of clinical experience in acupuncture practice. Interventions will be conducted according to STRICTA and ICH-GCP guidelines. To ensure the consistency of skill of various acupuncturists, two to three training workshops will be provided to acupuncturists who have participated in the study prior to the commerce of the study. The acupuncture experts panel of School of Chinese Medicine, The Chinese University of Hong Kong, will provide technique training on the selection of acupuncture points and needling techniques. The workshops will be videotaped for education purpose.

## 6. Treatment Principle and Acupuncture Point Selection

Based on TCM theory, the pathogenesis of dysphagia after stroke is due to the insufficiency of the liver and kidney, the stagnant qi and blood that fail to nourish the tongue, and pathogenic factors, notably wind and phlegm, that further obstruct the meridians and the throat, resulting in swallowing difficulty. The participants will be further classified into two subtype patterns by registered Chinese Medicine Practitioner involved in the study, that is, (1) pathogenic wind phlegm syndrome and (2) liver yang rebellion syndrome. Those patients with dizziness and thin tongue coating belong to the liver yang rebellion syndrome, while those who are with greasy tongue coating belong to the pathogenic wind phlegm syndrome. All patients in acupuncture group will receive acupuncture at acupuncture points such as GB12 (Wan Gu, bilateral), GB20 (Feng Chi, bilateral), CV23 (Lian Quan), DU20 (Bai Hui), and LI4 (He Gu, bilateral) and HT5 (Tong Li, bilateral), Hangsang, Shanglianquan, and Lower 2/5 of the motor zone of scalp acupuncture.

Besides the above regular acupuncture points, additional acupuncture points will be used for patients' clinical syndrome subtypes. These additional points include ST40 (Fenglong, bilateral) for pathogenic wind phlegm subtype and LR3 (Taichong, bilateral) for liver yang rebellion type.

## 7. Manipulation Procedure

Sterile disposable stainless steel needles of various lengths and diameters will be used in the study. The standardized needling manipulation methods on different acupuncture points are described as follows: GB12, GB20 (a depth of 0.8–1.2 cun with the needle tip pointing towards the throat will be punctured, and needles will be rotated with reinforcing techniques); CV23 (about 1.0 cun towards the root of the tongue will be inserted obliquely, and mild lifting and thrusting technique will be applied for about 1 min); DU20 (at an angle of 30 degrees, angle to a depth of 0.5 to 0.8 cun will be inserted obliquely, and the needle will be rotated about 30 seconds before withdrawing the needle); LI4 (puncture perpendicularly for 0.5 to 1.0 cun, and apply for 30 seconds reinforcing technique with the lifting and thrusting movement); HT5 (a depth of 0.5 cun will be punctured perpendicularly, reinforcing technique with the lifting and thrusting movement will be applied for 30 seconds); Hangsang (a needle of 75 mm in length will be applied to prick the posterior wall of the throat at the depth of about 0.15 cun for 4 times without retaining the needle); Shanglianquan (a depth of 1.0–1.5 cun towards the root of the tongue will be inserted obliquely, and mild lifting and thrusting technique will be applied for 30 seconds); Lower 2/5 of the motor zone of scalp acupuncture (the needle will be inserted transversely at an angle of 15 degrees to the skin surface and push the needle to about 1.0 cun in depth, and twirling method will be applied for about 30 seconds); ST40 (the needle will be punctured perpendicularly for 1.5 cun, and reducing technique by lifting and thrusting the needle will be applied); LR3 (the needle will be inserted perpendicularly for 0.5–1.0 cun, and reducing techniques by lifting and thrusting the needle for 30 seconds will be applied) (Table 1).

The acupuncture treatment will be administered once every two days, three times a week, and the whole treatment period will last for 2 months. A total of 24 acupuncture treatment sessions will be carried out on each patient.

Participants who are allocated to nonacupuncture controlled group will receive routine care without acupuncture treatment.

## 8. Primary Outcome

Royal Brisbane Hospital Outcome Measure for Swallowing (RBHOMS) is a valid bedside scale for monitoring difficulties in daily swallowing function based on clinical indicators of swallowing, but not on texture-modified diets [20]. RBHOMS is a 10-point ordinal scale that ranges over four stages of oral intake: stage A, nil by mouth (levels 1–3); stage B, commencing oral intake (level 4); stage C, establishing oral intake (levels 5–7), and stage D, maintaining oral (levels 8–10). Both acupuncture group and nonacupuncture control group patient will be conducted three times RBHOMS, with one being conducted at baseline, one at the end of treatment (2 months after inclusion), and the last one at the end of follow-up period (2 months after treatment completion). All RBHOMS will be conducted by a registered speech therapist blinded to group allocation.

## 9. Secondary Outcomes

The secondary outcome measures include functional oral intake scale [21], swallow quality-of-life questionnaire in Chinese version [22], BMI [7], and adverse event report.

Functional oral intake scale, or known as swallow function scores, is a 7-point scale that describes the severity of swallowing dysfunction.

Swallow quality-of-life questionnaire in Chinese version consists of 12 items. The items covered all physical and psychosocial impact of the patients. Swallow quality-of-life questionnaire in Chinese version is chosen as secondary outcome because it is as sensitive, valid, and reliable as original version [22].

All adverse events as defined by the Ethics Committee will be fully recorded on the Adverse Event Page of the Case Report Form.

All functional oral intake scale, swallow quality-of-life questionnaire in Chinese version, and BMI will be assessed at baseline (before the intervention), the end of treatment (2 months after inclusion), and the end of follow-up period (2 months after treatment completion).

## 10. Sample Size Estimation

The primary outcome measure is Royal Brisbane Hospital Outcome Measure for Swallowing (RBHOMS), which is a 10-point ordinal scale that ranges over four stages of oral intake from stage “A” (nil by mouth) to “D” (maintaining oral intake). The sample size is estimated to compare the proportion of those who are rated at stage “D.” Based on our previous clinical study [23], we estimate that 60 participants in each group will be needed to detect 20% difference of the proportion between the treatment and control group with a false positive error of 5% and power of 80%. To compensate for dropouts during treatment period, we inflate this value by 15% and hence a total of 140 patients will be recruited, with 70 each in the treatment and control group.

## 11. Data Analysis

All analyses will be conducted according to the intention-to-treat principle. Descriptive statistics will be computed for each of the analyzed variables. For the primary outcome, chi square test will be used. All continuous variables such as swallowing time, swallow quality-of-life, and BMI will be analyzed using *t*-test where appropriate. Swallow function scores will be analyzed using Mann–Whitney *U* test. The possible relationship between some baseline data and main outcomes will be explored using multivariable analysis. All statistical tests will be two-sided, and *p* < 0.05 is considered statistically significant. The statistical software of SPSS (SPSS, SPSS Inc., Chicago, USA) version 20 will be used for the analysis.

## 12. Discussion

This pilot study is designed to evaluate the effectiveness and the safety of acupuncture for poststroke dysphagia. As the purpose of our pilot study is to assess the effectiveness and safety of acupuncture in managing poststroke dysphagia within the healthcare setting in Hong Kong, the pragmatic design is deemed appropriate. A routine supportive and rehabilitative treatment group without acupuncture is designed as a control group instead of sham acupuncture, as it is unrealistic to ask the stroke patients to receive sham acupuncture.

Due to the safety consideration and feasibility, RBHOMS is to be selected as the primary outcome. RBHOMS is widely used as bedside assessment in dysphagia patient. It is a noninvasive investigation, and the time required to perform this test is about 10 minutes. As it will not bring too much burden to the patients, it is anticipated that the compliance of patients in the control arm would be high.

The functional oral intake scale, coupled with swallow quality-of-life questionnaire in Chinese version, BMI, and adverse events report are to be used as secondary outcome. The functional oral intake scale is an ordinary rate scale to assess the swallowing function of a patient. It is an auxiliary instrument to assess swallowing function of the patients. Previous study [20] illustrated that swallow quality-of-life questionnaire in Chinese version is a valid, effective, and sensitive tool to assess the physical and psychosocial impact in dysphagia patient of Chinese population.

The strength of our pilot study design is the pragmatic approach. This study mimics the real-world clinical situation based on Hong Kong healthcare setting. Standard acupuncture treatment protocol is obtained by the consensus of our acupuncture expert panel. Although, in real-world setting, most acupuncturists will individualize their acupuncture points selection and manipulation based on TCM theory. However, considering the consistency and replicability of the trial, a standardize acupuncture treatment protocol is chosen. Even that standardized regular acupuncture points are adopted, all enrolled participants will be classified into two subtype syndromes according to diagnosis criteria and different additional acupuncture points will be punctured individually.

Blinding is a crucial measure to assure the quality of the trial. All outcome assessor and statistician will be kept blind throughout the whole process. We will minimize the interaction between the patients and outcome assessors. Neither the RBHOMS assessor nor the staff who administer the questionnaire knows the group allocation of the participants.

One main limitation of this study is lack of randomization. Randomization is a key procedure to minimize the selection bias and ensure the comparability of all parameters between the intervention group and control group. However, concerning the timeframe and budget of our pilot study, nonrandomization approach will be utilized. To reduce the selection bias of our study, subjects will be recruited from different sources, including hospitals, speech therapy outpatient clinics, and Chinese medicine clinics. Moreover, all the prognostic factors and baseline characteristics of participants such as gender, age, occupation, life style, and onset time of stroke will be controlled in both acupuncture group and nonacupuncture control group. All data will be collected to examine the baseline comparability and explore the possible sources of dissimilarity and make proper statistical adjustment later.

As for all clinical trials, subject recruitment is one of the most critical factors for the trial's successful implementation. In our study, various venues and approaches will be utilized to augment the subject recruitment process. Free Chinese medicine consultation will be provided to control arm participants as an incentive. In addition, we will also hold promotional talks in the Chinese medicine clinics to facilitate the recruitment process. Moreover, we may also place advertisements on local newspapers and contact the stroke patients association to boost subject recruitment if recruitments from hospitals and Chinese medicine clinics turn out to be undesirable.

Nonetheless, most of potential subjects of this study are stroke sufferers. They always have other complications, such as immobility. In order to reduce the dropout rate and to enhance the compliance of patients, some treatment sessions may be conducted at affiliated mobile clinics.

This pilot study is the first large scale, multicenter controlled trial focusing on the acupuncture for poststroke dysphagia, which is one of the common complications of stroke patient. Although there is some limitation of the study, the findings of this project are able to provide an early evidence for the effectiveness of acupuncture treatment in enhancing swallowing function and life quality of poststroke dysphagia patients.

## Figures and Tables

**Table 1:** Locations, indications, and manipulations of the acupoints selected for the project.

Points	Location	Indications	Manipulation
GB20 (Feng Chi)	In the depression between m. sternocleidomastoideus and m. trapezius, level with Fengfu (DU 16) point (on the Du meridian, 1 cun above the posterior hairline). [If the hairline is not easily determined, the level of bilateral earlobe tip connection will be used.]	Headache, stiff neck, red and painful eyes, nose bleeding, tinnitus, stroke, swallowing difficulty, deviated mouth, and eyes.	Puncture at a depth of 0.8–1.2 cun with the needle tip pointing towards the throat. After obtaining needling sensation, rotate the needles with reinforcing techniques (rotating 180°–270° at 30 times/min for 30 seconds) till the needling sensation radiating to the whole tongue and throat.

GB12 (Wangu)	On the head, in the depression posterior-inferior to mastoid process.	Headache, neck stiffness, toothache, facial palsy.	Similar to GB20

CV23 (LianQuan)	With the patient's head slightly tilting backward, the point is directly above the Adam's apple and at the midpoint of the upper border of the hyoid bone.	Swelling and pain in the tongue, excessive salivation, stroke with stiffness of the tongue, sudden loss of voice, difficulty in swallowing.	Insert obliquely for about 1.0 cun towards the root of the tongue, followed by applying mild lifting and thrusting technique for about 1 min till soreness and distending sensation (de qi) is felt in the tongue and throat.

Shanglianquan	1 cun above CV23 (with the patient in a sitting position and the patient's head slightly tilting backward, the point is directly above the Adam's apple, and on the midpoint of the upper border of the hyoid bone).	Alalia, sore throat, difficulty in swallowing, difficulty in speech	Insert the needle obliquely to a depth of 1.0–1.5 cun towards the root of the tongue. Then followed by mild lifting and thrusting the needle for around 30 seconds until soreness and distending sensation is felt in the tongue and throat.

Hangsang	On the posterior wall of the throat.	Sore throat, difficulty in speech.	Patient is asked to open the mouth, with the tongue pressed by a tongue depressor to expose the posterior wall of the throat clearly. Then a needle of 75 mm in length will be applied to prick the posterior wall of the throat at the depth of about 0.15 cunfor 4 times. After the needle is withdrawn, the patient is asked to move the tongue in and out for 10 times.

Lower 2/5 of the motor zone of the scalp	The motor zone is a line connecting the following two points: the first is 0.5 cm posterior to the midpoint of the anteroposterior midline over the vertex; the second is the intersection of the lateral eyebrow-occiput line with the anterior border of the hairline at the temple. The motor zone can be divided into 5 portions.	Facial paralysis, swallowing difficulty.	Insert the needle transversely at 15° angle to the skin surface and push the needle to about 1.0 cun in depth, then use twirling method (rotating 180°–270° about 200 times/min) to manipulate the needle for about 30 seconds.

DU20 (Bai Hui)	7 cun above the midpoint of the posterior hairline, or at the junction of the midline at the vertex with the line joining the two ear apexes.	Headache, tinnitus, vertigo, stroke with difficulty to speak.	Puncture obliquely at an angle of 30 degrees to a depth of 0.5 to 0.8 cun, then use rotating motion (rotating 180°–270° at about 200 times/min) to manipulate the needle for about 30 seconds before withdrawing the needle.

LI4 (HeGu)	On the dorsum of the hand, between the first and second metacarpal bones, approximately in the middle of the second metacarpal bone on the radial side.	Swollen and sore throat, headache, toothache, deviated mouth and eye, fever, delayed labour.	Puncture perpendicularly for 0.5 to 1.0 cun, then apply for 30 seconds reinforcing technique with the lifting and thrusting movement (strong thrusting and gentle lifting for short duration with small amplitude and low frequency).

HT5 (Tongli)	1 cun above the shenmen point, which is at the medial end of the transverse crease of the wrist, in the depression on the radial side of the tendon of the flexor carpi ulnaris.	Painful and swollen throat, sudden loss of voice, stiff tongue with difficulty in speech.	Puncture perpendicularly to a depth of 0.5cun, then apply reinforcing technique with the lifting and thrusting movement for 30 seconds.

ST40 (Fenglong)	8 cun above the external malleolus, 2 cun lateral to the anterior crest of the tibia.	Headache, cough and stroke with phlegm as the pathogenic cause.	Puncture perpendicularly for 1.5 cun, then apply reducing technique by lifting and thrusting the needle (gentle thrusting and strong lifting with large amplitude and high frequency) 30 seconds.

LR3 (Taichong)	On the dorsum of the foot, in the depression distal to the junction of the 1st and 2nd metatarsal bones.	Headache, dizziness, blurred vision, deviated mouth, stroke.	Puncture perpendicularly for 0.5–1.0 cun, then apply reducing techniques by lifting and thrusting the needle for 30 seconds.

KI3 (Taixi)	At the depression between the medial malleolus and the tendon calcaneus.	Sore throat, deafness, coughing blood, impotence, frequent urination and lumbar pain.	Insertion perpendicularly for 0.5–1.0 cun, then apply for 30 seconds reinforcing technique with the lifting and thrusting movement.

UB23 (Shen Shu)	Level with the lower border of the spinous process of the 2nd lumbar vertebra, 1.5 cunlateral to the posterior midline.	Blurred vision, deafness, tinnitus, edema and lower back pain.	Straight insertion for 0.5–1.0 cun, then apply for 1 min reinforcing technique with the lifting and thrusting movement.

## Abbreviations

BMI: Body mass index
RBHOMS: Royal Brisbane Hospital Outcome Measure for Swallowing
TCM: Traditional Chinese medicine.

## Additional Points

*Trial Status*. This trial is currently ongoing.
